# Single Point Mutation and Its Role in Specific Pathogenicity to Reveal the Mechanism of Related Protein Families

**DOI:** 10.1128/spectrum.00923-22

**Published:** 2022-10-10

**Authors:** Ning Liu, Xue Wang, Qiang Shan, Shuxian Li, Yanan Li, Bingxin Chu, Jiufeng Wang, Yaohong Zhu

**Affiliations:** a Department of Veterinary Clinical Sciences, College of Veterinary Medicine, China Agricultural University, Beijing, China; Hubei University of Medicine

**Keywords:** pyolysin, cholesterol-dependent cytolysin, mutation, *Trueperella pyogenes*, protein structure

## Abstract

Pyolysin (PLO) is secreted by Trueperella pyogenes as a water-soluble monomer after forming transmembrane β-barrel channels in the cell membrane by binding cholesterol. Two significantly conserved residues at domain 1 of PLO are mutated, which provides novel evidence of a relationship between conformational change and interaction with the cell membrane and uncovers the pore formation mechanism of the cholesterol-dependent cytolysin (CDC) family. Moreover, PLO is a special member of the CDCs, which the percentage of sequence identities between PLO and other CDC members is from 31% to 45%, while others are usually from 40% to 70%. It is important to understand that at very low sequence identities, models can be different in the pathogenic mechanisms of these CDC members, which are dedicated to a large number of Gram-positive bacterial pathogens. Our studies, for the first time, located and mutated two different highly conserved structural sites in the primary structure critical for PLO structure and function that proved the importance of these sites. Together, novel and repeatable observations into the pore formation mechanism of CDCs are provided by our findings.

**IMPORTANCE** Postpartum disease of dairy cows caused by persistent bacterial infection is a global disease, which has a serious impact on the development of the dairy industry and brings huge economic losses. As one of the most relevant pathogenic bacteria for postpartum diseases in dairy cows, Trueperella pyogenes can secrete pyolysin (PLO), a member of the cholesterol-dependent cytolysin (CDC) family and recognized as the most important toxin of *T. pyogenes*. However, the current research work on PLO is still insufficient. The pathogenic mechanism of this toxin can be fully explored by changing the local structure and overall function of the toxin by a previously unidentified single point mutation. These studies lay the groundwork for future studies that will explore the contribution of this large family of CDC proteins to microbial survival and human disease.

## INTRODUCTION

The cholesterol-dependent cytolysins (CDCs) are a family of pore-forming toxins expressed by many Gram-positive pathogens (1) and specifically target eukaryotic cells through the absolute requirement for high concentrations of cholesterol in the target cells’ lipid membrane (2), assemble into large oligomeric β-barrel pore complexes (3), and are linked with the virulence mechanisms of a wide spectrum of pathogenic bacteria (4, 5). Cholesterol binding initiates significant secondary and tertiary structural changes in the monomers (6). After soluble CDC monomer binding to membranes, the monomers then interact with each other to form an oligomeric prepore, and then the prepore structure collapses vertically to penetrate into the membrane, becoming a transmembrane β-barrel pore (7, 8). This procedure is usually followed by significant conformational changes in the structure (4, 9–12). The other lipid components are dependent not only on membrane cholesterol but also on the presence of CDCs in the bilayer (2). Members of this family present high levels of homology in their primary structures (40 to 70%) and in the crystal structures of their soluble monomers (13, 14). Emerging knowledge indicates that all eight major CDCs have high-affinity lectin activity that identifies glycans as candidate cellular receptors (15).

Pyolysin (PLO) is a member of the CDC family that consists of listeriolysin O, pneumolysin (PLY), suilysin, streptolysin O, intermedilysin (ILY), perfringolysin O (PFO), arcanolysin (14, 16–21), and others. However, the primary structure of PLO only presents a low level of homology with other members from 31% to 45% (21). Such a low homology greatly limits speculation of the function, structure, and pathogenic mechanism of PLO. Several key sequence motifs have been identified as characteristic of the CDCs as a family (3) and tie into the structure-function aspects of CDC activity (2). Studies are usually focused on these conserved motifs in domains 2, 3, and 4 after forming a membrane-binding interface that stably anchors the toxin to the membrane surface after interaction of the cholesterol recognition/binding motif (CRM) with cholesterol (22–24). However, little research has been done on the role of domain 1 after membrane recognition and binding.

The methods of studying PLO are also very limited (3, 25–28). We can predict the structure and function of PLO by studying other CDC family members. PLO, unlike intermedilysin (ILY) (29) from Streptococcus along with the human CD59 (hCD59)-specific cytolysin, specifically targets cholesterol to form pores (30). The cysteine residue required for thiol activation has been replaced with alanine (31), for which the impact of its function has remained enigmatic. In addition, amino acids from 123 to 166 at the N terminus of PLO are important for PLO activity, as monoclonal antibodies directed to these regions block hemolytic activity (30). This region locates within domain 1 of PLO, which its function and structure have not been studied sufficiently. Until now, little study has been performed investigating the function and structure of PLO.

In order to determine the structural sites that mediate the highly specific interaction of PLO with the cell membrane and the mechanism of PLO binding with cholesterol, we performed a detailed investigation on nonfunctional PLO variants that harbor a single point mutation, which are highly conserved in CDCs and are essential for cell membrane binding; the pathogenicity of these mutants was remarkably modulated. These different mutations can not only affect the interaction between toxins and red blood cell membrane (RBCM) but also affect the oligomerization ability of membrane-bound toxin molecules. Our studies also reveal the basis for the cholesterol-binding mechanism with PLO and provide an explanation of the cholesterol dependence of CDCs.

## RESULTS

### Recombinant mutants of PLO with a single point mutation.

**FIG 1:**
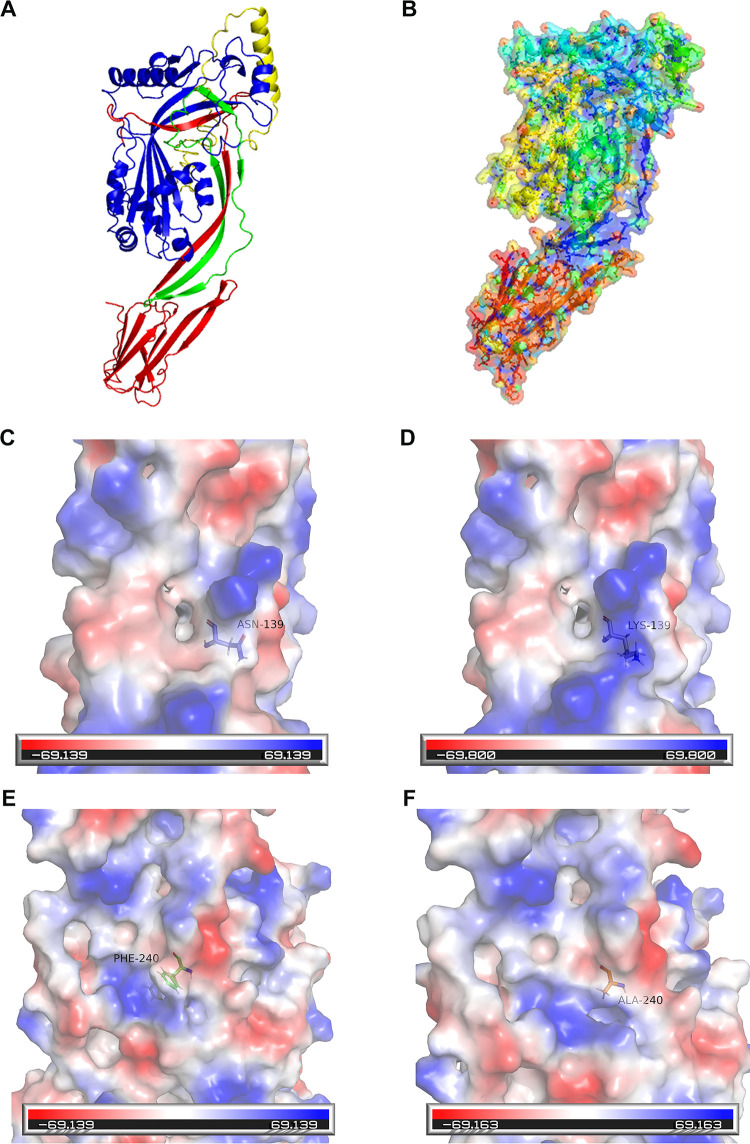
Three-dimensional structure of rPLO and locally enlarged 3D structure of the mutation sites. (A and B) The 3D structure of rPLO is presented in different colors. Each color represents one domain. (C and D) The locally enlarged 3D structures of residue 139. Asparagine was replaced with lysine. The mutation at this site can observe structural changes. (E and F) The locally enlarged 3D structures of residue 240. Phenylalanine was substituted with alanine. Structure changes with amino acid substitution.

In the procedures of cloning, expression, and purification of recombinant PLO (rPLO), two rPLO mutants (rPLO N139K and rPLO F240A) were accessed harboring a single point mutation (Fig. 1). The point mutation occurred from a change in the fusion PCR amplification process of the cloning when the nucleotide sequence was established. During the procedures of expression and purification, the PLO mutants used the same preparation process as that of the rPLO, and the solubility between the mutants and rPLO were not significantly different. All the proteins were quantified and qualified after purification by NanoDrop and sodium dodecyl sulfate-polyacrylamide gel electrophoresis (SDS-PAGE). In order to further explore the influence of structural differences between the mutants and rPLO, three-dimensional (3D) models (Fig. 1A and B) based on the homology structural template were introduced for comparison. The results indicate that both mutations of PLO did not lead to principal spatial conformational changes of secondary structure and tertiary structure of the protein (Fig. 1C to F). The structure of PLO shows that the selected mutant residues 139 and 240 are located in domain 1 of the 4 domains of PLO, which is rarely studied.

**FIG 2:**
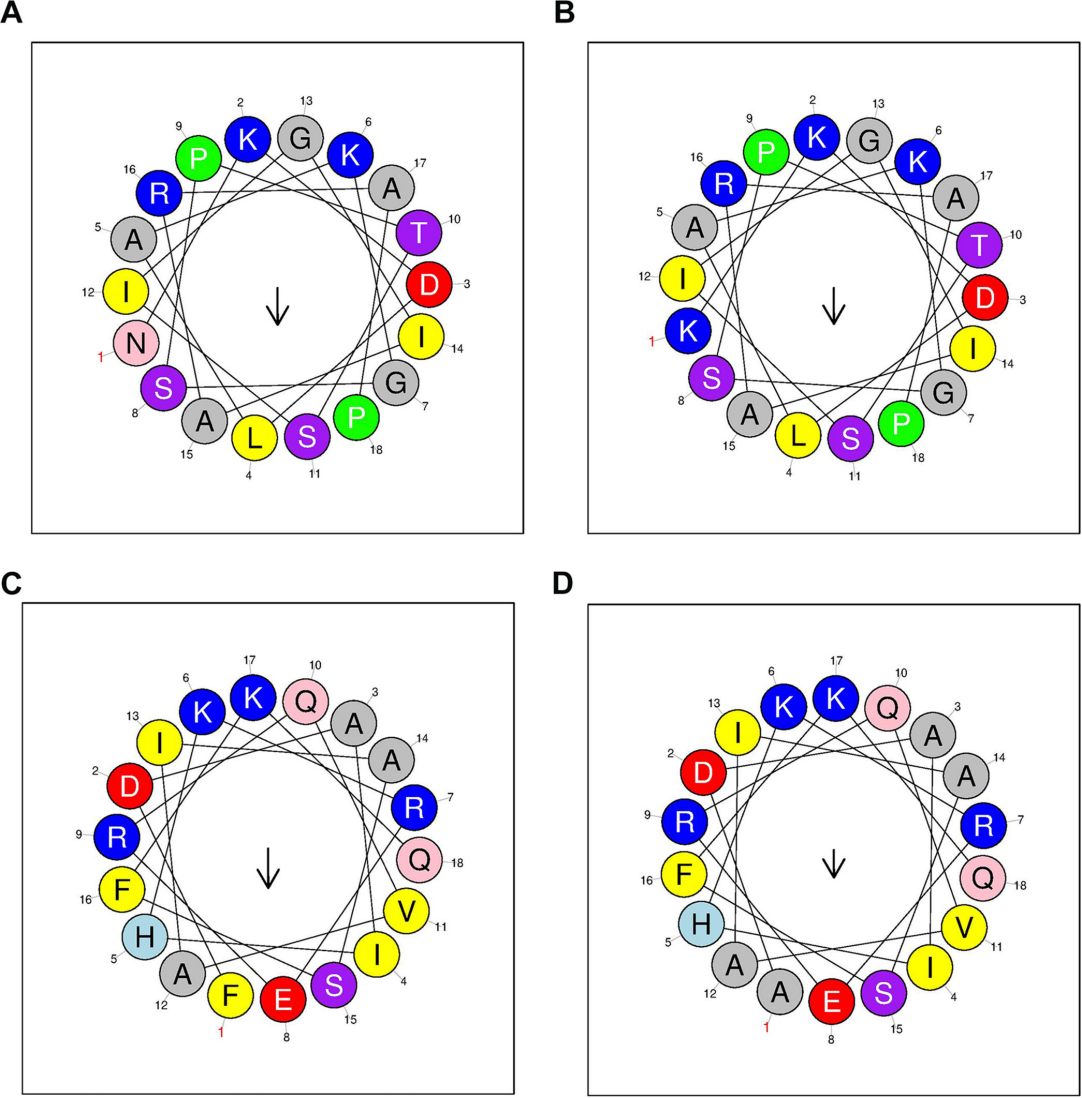
Hydrophilic analysis of rPLO and its mutants. (A and B) A standard helical wheel diagram showing the distribution from residues 139 to 156 with or without the residue 139 mutation. (C and D) A standard helical wheel diagram showing the distribution from residues 240 to 257 with or without the residue 240 mutation. The arrow in the helical wheel indicates the direction of the hydrophobic moment.

We intercepted 18 amino acid sequences that started with substituted residues 139 and 240 as the N terminus for hydrophobicity analysis (Fig. 2 and Table 1). Notably, compared with rPLO (Fig. 2A and C), the hydrophobicity of rPLO N139K (Fig. 2B) and rPLO F240A (Fig. 2D) decreased. Interestingly, along with the visible differences of protein structure of residues 139 and 240 between PLO and the mutants, the hydrophobicity and hydrophobic moment of the mutants were decreased compared to rPLO by mutating a single residue. Moreover, compared with the asparagine and phenylalanine side chains, our results match with the decreased hydrophobicity of the lysine and alanine side chains, respectively. These data indicate that the rPLO N139K and rPLO F240A mutants may have substantial changes in membrane interactions at the molecular level of the PLO protein structure.

**TABLE 1:** Protein surface property analysis of different residues

Residues	N139	N139K	F240	F240A
Hydrophobicity (H)	0.194	0.172	0.198	0.116
Hydrophobic moment (μH)	0.243	0.236	0.260	0.182
Net charge (*z*)	2	3	2	2
Polar residues +GLY (*n*/%)	10/55.56	10/55.56	10/55.56	10/55.56
Nonpolar residues (*n*/%)	8/44.44	8/44.44	8/44.44	8/44.44

### The hemolytic activity and cytotoxicity of the rPLO mutants at residues 139 and 240.

**FIG 3:**
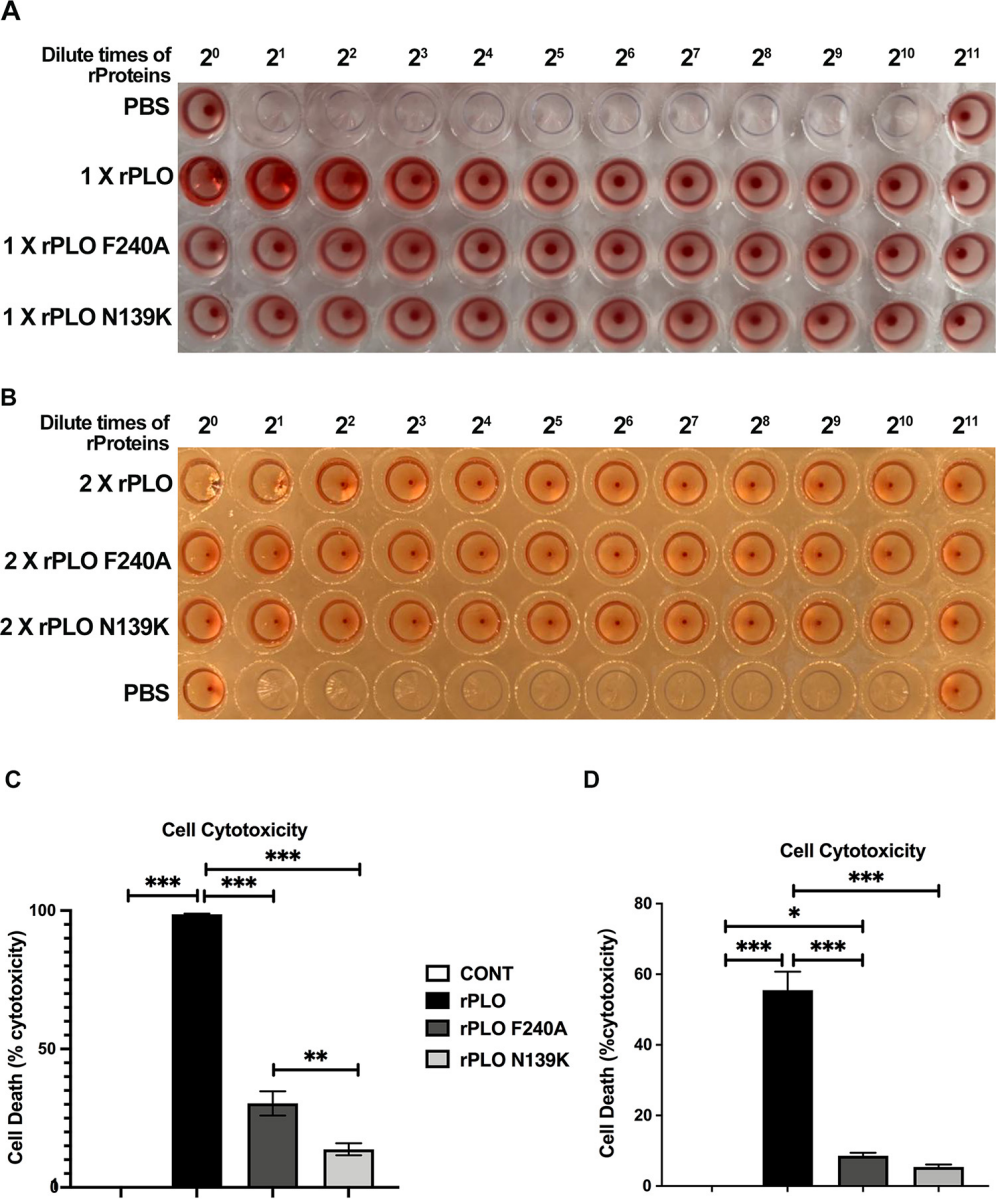
Hemolytic activity and cytotoxicity of rPLO and its mutants. (A) The hemolytic activity of the initial protein concentration of 100 μg/mL. The proteins were diluted 2-fold with PBS to 2^11^. PBS was applied as a negative control. After a 30-min static incubation at 37°C, blood cells aggregated at the bottom of the nonhemolytic samples. No blood cell aggregation occurred at the bottom of the hemolytic samples. (B) The initial protein concentrations were increased to 200 μg/mL. The observation of protein hemolysis was followed. (C and D) Cytotoxicity of rPLO and its mutants was measured by LDH. A comparison of the cytotoxicity of rPLO and its mutants on cells incubated for 30 min is shown. The data are expressed as a percentage. The mean ± SEM values of the data are shown from three independent repeats (**, *P* < 0.01, ***, *P* < 0.001).

Hemolytic activity is an important characteristic of the CDC family. In the present study, rPLO exhibited strong hemolytic activity against blood cells (Fig. 3A). Neither of the replacements was tolerated; a significant reduction in hemolytic activity was detected in both substitutions. Namely, rPLO N139K and rPLO F240A did not cause measurable hemolytic activity in blood cells. Even if we doubled the protein concentration, neither mutant showed detectable hemolytic activity compared with rPLO, of which hemolytic activity is significantly reduced (Fig. 3B). The results indicate that N139K and F240A mutants critically affect the pore-forming capacity of PLO on the cell membrane.

Asn-139 and Phe-240 are two conserved sites on PLO. HeLa cells (Fig. 3C) and bovine endometrial epithelial cells (BEECs) (Fig. 3D) were both used to detect the cytotoxicity of rPLO and its mutants, respectively. Substitutions of the PLO at residues 139 and 240 with lysine and alanine significantly decreased its cytotoxicity (*P* < 0.01), whereas substitution of residue 240 with alanine had less of an effect on cytotoxicity. In Fig. 3D, supplementation of rPLO N139K did not show a statistically significant difference compared with control cells. HeLa cells are more sensitive to proteins than BEECs. We considered whether the hydrophobicity of mutated amino acid residues might be an artifact of PLO-mediated damage to the cell membrane that induced cell lysis. The hydrophobicities of asparagine and phenylalanine residues are lower than those of lysine and alanine. The rPLO F240A and rPLO N139K mutants both had significantly reduced cytotoxicity compared to rPLO. Therefore, these results indicate that cytotoxicity may be responsible for the hydrophobicity of the residue substitutions.

### Membrane binding and oligomerization of the PLO mutants.

**FIG 4:**
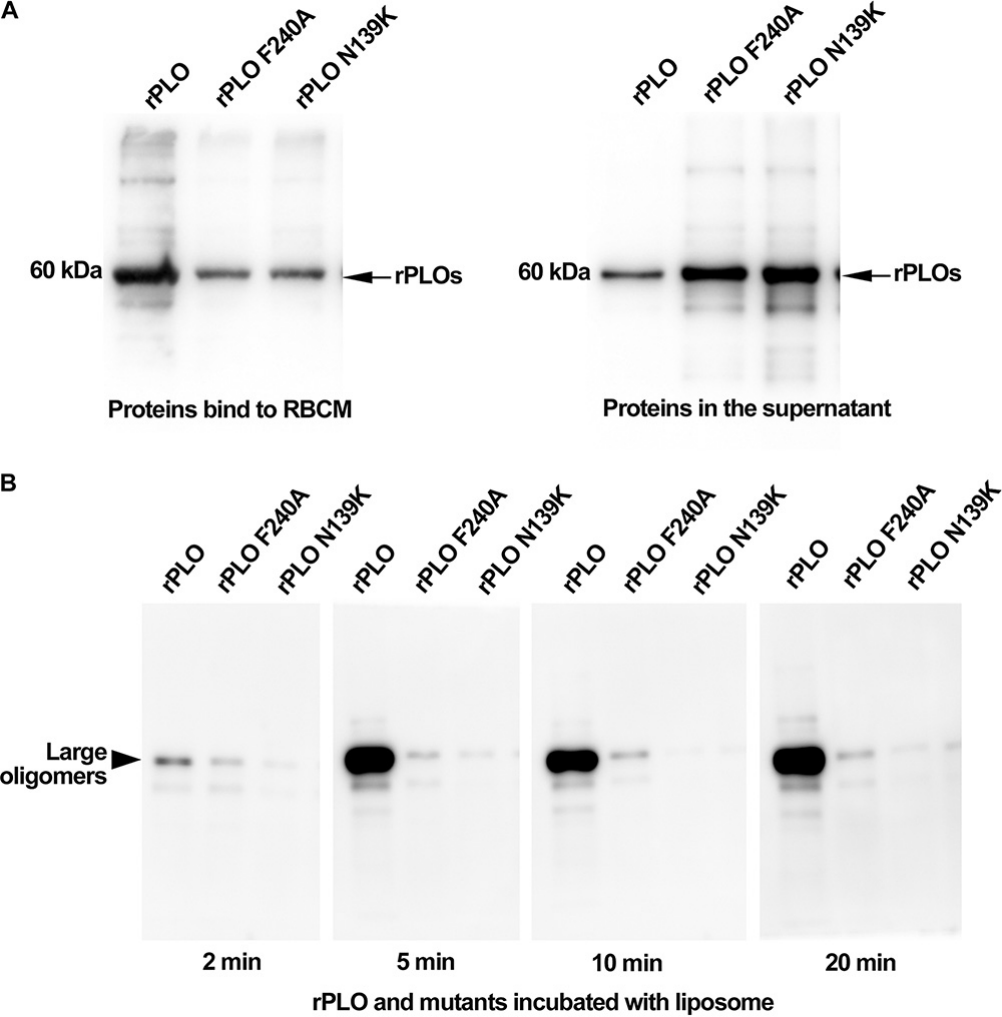
Membrane binding ability and oligomerization capacity of rPLO and its mutants. (A) The proteins bound to RBCM in precipitate and unbound to the RBCM in supernatants, respectively. The molecular weight of the proteins is about 60 kDa. (B) The ability of proteins to form large oligomers. rPLO and its mutants incubated with liposomes for 2 min, 5 min, 10 min, and 20 min was assessed by Western blotting.

To discover the mechanism of how mutations at residues 139 and 240 reduce the hemolytic capacity of the PLO toxin, we analyzed the binding and oligomerization activities of rPLO and the two mutants to the RBCM (Fig. 4). We observed rPLO and the mutants targeting the RBCM by immunoblotting using an anti-His-tagged antibody (Fig. 4A). Quantification of Western blotting results indicated that the F240A and the N139K mutants maintained approximately less than one-third of the binding ability of rPLO. The binding abilities of F240A and N139K had no visible difference, which is basically in accordance with the hemolytic ability data of those two mutants. These data revealed that due to a single point mutation at residue 139 or 240, the interaction of PLO with the RBCM diminished to a valuable extent. However, this conclusion is not completely convincing.

CDC family members, such as perfringolysin O (PFO), polymerize large SDS-resistant prepore complexes possessing monomers (13, 32). Western blotting analysis showed that rPLO formed large oligomers after incubation with cholesterol-based liposomes. However, the mutations notably impaired the ability to form large oligomers (Fig. 4B). rPLO started to form large oligomers at 2 min after incubation with liposomes, and there was a significant difference compared to mutants at 5 min. The oligomerization ability of the N139K mutant was almost lost, while the oligomerization ability of the F240A mutant maintained a little. Our data show that the F240A mutant can also form different-sized oligomers, but they are not obvious compared to the oligomers of rPLO. The N139K mutant completely lost the ability to form oligomers of other sizes.

### The effect of mutation on pore-forming ability.

**FIG 5:**
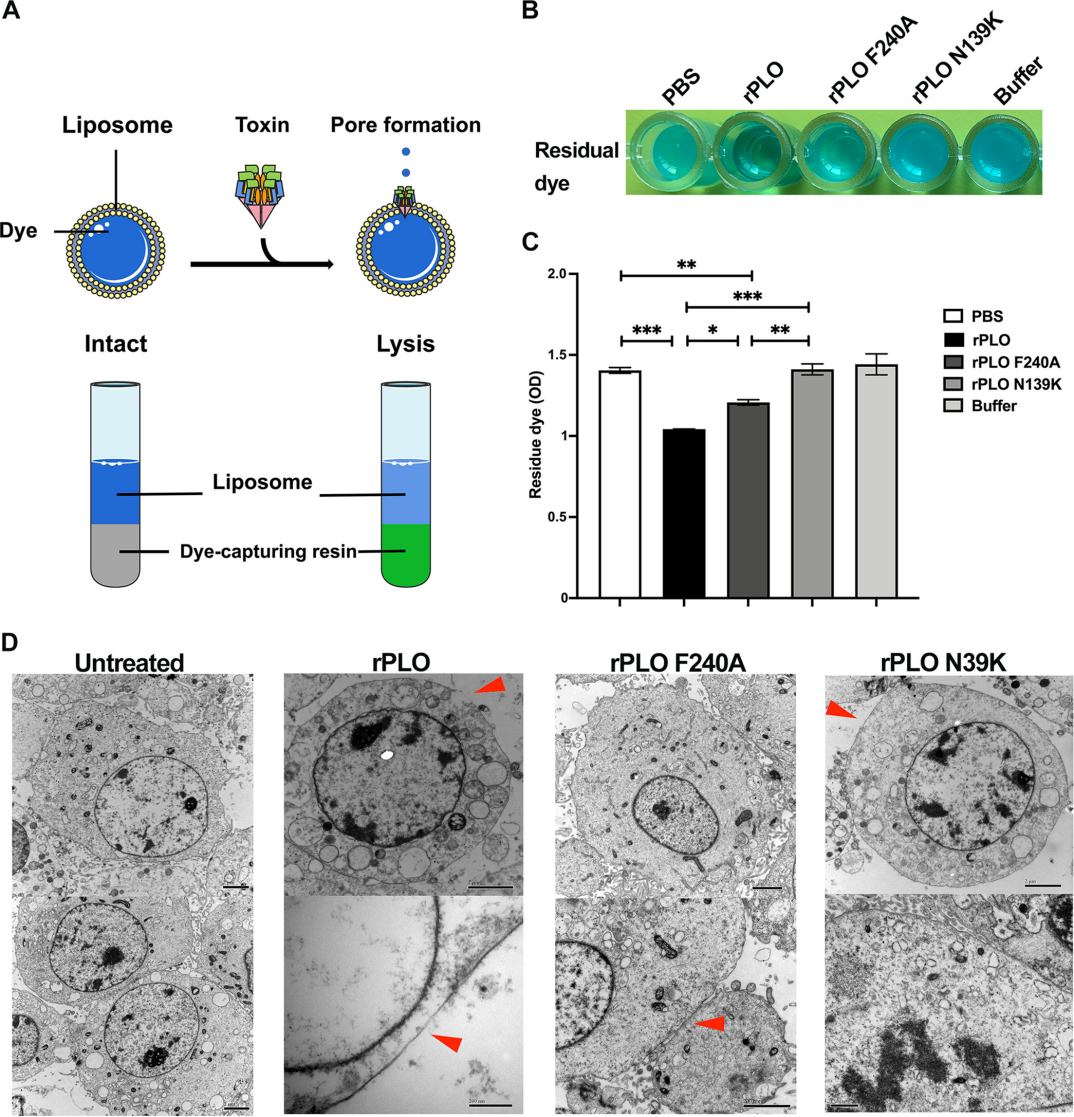
Demonstration of protein pore-forming ability. (A) Schematic illustration of toxin-induced liposome disruption and dye leakage. (B) Colorimetric analysis of untreated liposomes treated with PBS, rPLO, rPLO F240A, rPLO N139K, and buffer. (C) The absorbance (OD) of residual dye after treatment as in B. (D) Transmission electron micrographs showing pore formation of proteins on the cell membrane.

To explore the molecular basis for the inability of rPLO mutants to form pores, the lipid bilayer was introduced by formulating liposomes composed of lecithin and cholesterol. We hypothesized that the mutations on residues 139 and 240 destroy the oligomerization and pore-forming ability of the PLO toxin by destabilizing the plasma membrane. An internal aqueous methylene blue dye was wrapped in liposomes, which, when released, can be captured by resins (33) (Fig. 5A). The addition of rPLO, but not rPLO F240A, rPLO N139K, buffer, or phosphate-buffered saline (PBS), caused the release of dye from the liposomes (Fig. 5B and C). The dye leaked at the highest level when rPLO was incubated with liposomes. Furthermore, incubation of rPLO N139K or buffer with liposomes did not show dye leakage compared to incubation of PBS with liposomes. Incubation of rPLO F240A with liposomes caused a slight dye leakage.

Under transmission electron microscopy (TEM), we observed that rPLO formed pores in the cell membrane (Fig. 5D). The ability of rPLO to bind cholesterol and form pores in the membrane indicated that this type of toxin could kill not just epithelial cells from a single species. Not surprisingly, PLO mutations at residues 139 and 240 inhibited the ability to form pores. However, rPLO F240A still retained a limited pore-forming ability, which is likely to be closely related to the formation of large oligomers of proteins and insertion into the cell membrane surface. This is also consistent with our findings.

### Mutation of PLO attenuates virulence and allows host tolerance of PLO.

**FIG 6:**
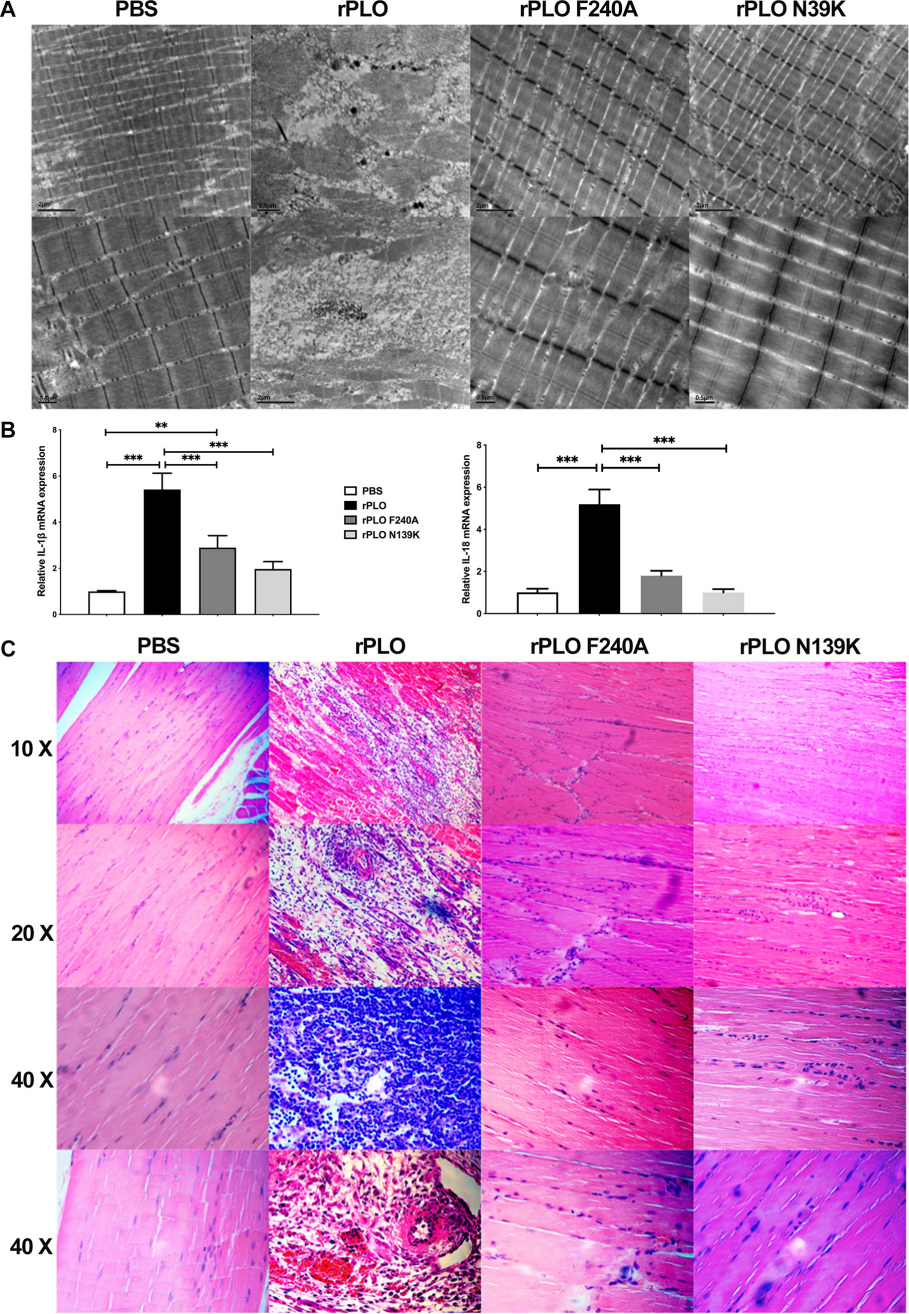
Protein damage to mouse tissue. (A) The extent to which mouse tissues are damaged by the protein. (B) Detection of protein-induced IL-1β and IL-18 inflammatory responses in mouse tissues. (C) H&E staining on mouse tissues after protein treatment.

Because PLO is typically considered a bacteriocin of extracellular pathogens, we attempted to investigate the significance of eliminating pore-forming ability in an *in vivo* infection hypothesis. We performed an experiment in a mouse model of rPLO, rPLO F240A, rPLO N139K, or PBS infection. TEM data indicated a conspicuous difference in the outcome of infection, with all rPLO-infected mice succumbing to infection at 72 h, with muscle tissue destroyed (Fig. 6A). Conversely, the condition of muscle tissues of rPLO F240A- and rPLO N139K-infected mice did not show striking signs of damage compared to the PBS-treated group. Moreover, because PLO is a key promoter of host inflammation, tissue injury, morbidity, and mortality (34), we investigated the inflammatory response and structural destruction in muscle tissue of mice infected with rPLO, the two mutants, and PBS. Mice infected with rPLO F240A and rPLO N139K had significantly reduced muscle inflammation and less structural destruction, as evidenced by decreased expression of the inflammatory cytokines interleukin-1β (IL-1β) and interleukin-18 (IL-18) compared to mice infected with rPLO (Fig. 6B). Histopathological analysis of mouse muscle tissues displayed similar outcomes (Fig. 6C). Introduction of rPLO into the muscle tissues triggered higher inflammatory responses than introduction of rPLO F240A and rPLO N139K.

### Crystal structure and molecular docking of rPLO mutants.

The roles of domains 2, 3, and 4 of PLO in mediating hemolytic activity, regulating oligomerization, and forming pores have been adequately studied (22). However, the function of domain 1 is still unclear as is how domain 1 cooperates with the other 3 domains. Residue 139 is a highly conserved asparagine in some CDC members, including PLO, thiol-activated cytolysin, and pneumolysin (PLY) from Streptococcus pneumoniae. In addition, residue 139 of listeriolysin O (LLO) from Listeria monocytogenes, perfringolysin O (PFO) from Clostridium perfringens, streptolysin O (SLO) from group A Streptococcus, and tetanolysin O (TLO) from Clostridium tetani are all a strikingly conserved aspartic acid, which shares a similar hydrophobicity and molecular weight as asparagine. Additionally, residue 240 in PLO is striking conserved with all of these toxins except for TLO, which incorporates a leucine. Such special sites make them important to PLO.

**FIG 7:**
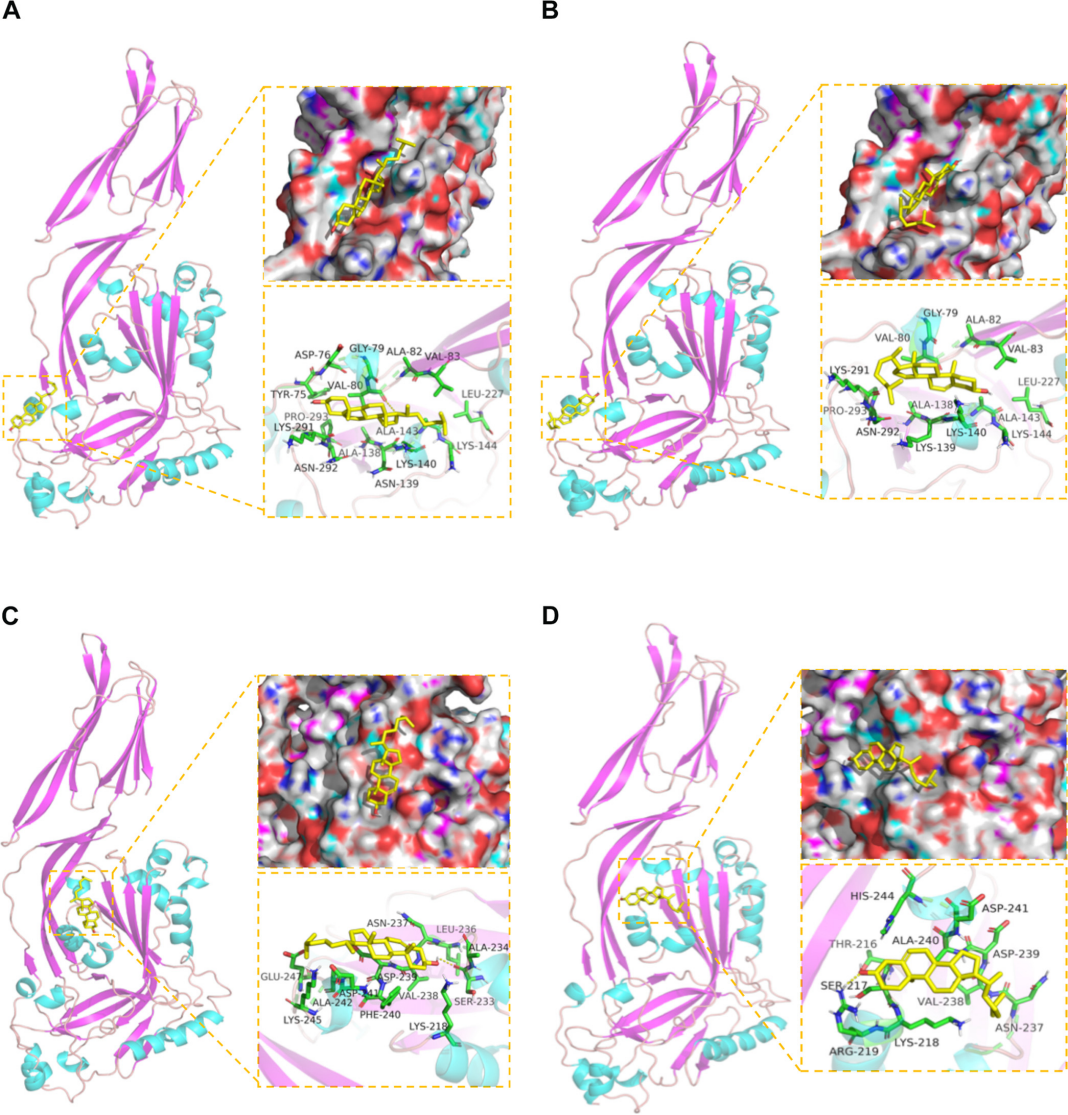
The binding mode of rPLO with cholesterol small molecule. (A and C) The 3D structure of rPLO. The surface of the active site and the detailed binding mode of rPLO at residues 139 and 240 are enlarged. Cholesterol small molecule is rendered in yellow. The yellow dash represents hydrogen bond distance or π-stacking. (B and D) The 3D structure of rPLO N139K and rPLO F240A. The surface of the active site and the detailed binding mode of rPLO mutants. Cholesterol small molecule is rendered in yellow. The yellow dash represents hydrogen-bond distance.

To assess the interaction of the protein-molecule complex, model structures of rPLOs and cholesterol were developed by protein docking prediction, and we calculated the binding energies for the complexes (Table 2). The calculated binding energy of rPLO was the lowest, while the binding energy of rPLO N139K was the highest. The lower the binding energy, the stronger the combination of protein-molecule complexes. This means that rPLO N139K has the weakest binding strength to small molecules. These data are consistent with previous results in this study. Furthermore, the overall and enlarged 139- and 240-residue conformational structures of rPLO, rPLO F240A, and rPLO N139K are shown in Fig. 7A to D, respectively. By comparison, we can find that the mutations have changed the way of these sites binding to cholesterol. This different matching method may be another key factor that affects the toxicity of PLO and even all CDC members.

**TABLE 2:**
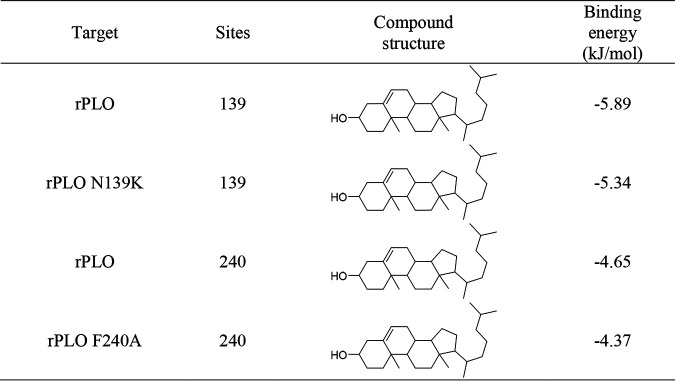
Target protein docking results for compounds

## DISCUSSION

Although a large number of reports have confirmed that members of the CDC family can interact with host cells in a variety of ways (35, 36), the innate cell membrane binding ability may prompt those bacteriocins to take priority to attach to the cell membrane (37). Therefore, instead of considering these toxins as ligands to study their characteristics, more attention should be paid to the adverse effects of their pore-forming ability on the host. Thus, more investigations devoted to studying the mechanism of pore formation of these toxins possess clinical application value.

In this study, we have designed two PLO mutants, which carry a single-residue substitution in the nonregular coil in domain 1 of PLO. Our data show that a single-residue mutation of N139K in PLO terminates the membrane pore-forming ability, while mutation of F240A causes restricted pore-forming ability compared to rPLO, indicating that the asparagine at residue 139 and the phenylalanine at residue 240 are critical for pore-forming ability in PLO. From our present experimental results, the hemolytic activity, membrane-binding ability, oligomerization ability, pore-forming ability, and inflammation ability of rPLO N139K and rPLO F240A are significantly lower than those of rPLO, but these effects of rPLO F240A have not been fully achieved to the extent of rPLO N139K loss. To explain the difference, we need to go through two aspects in protein structure, binding energy and the matching degree of small molecules and protein “pockets.”

Changes in the structure of domain 1 can cause the disturbing effect of pore-forming ability of PLO (22, 38). Thermodynamically, the strength of the association between a ligand molecule and its target receptor is measured by the standard binding free energy (39). The results of the binding energy of these three proteins show that rPLO binds to small molecules most tightly, while rPLO N139K binds to cholesterol molecules unstably. Cholesterol molecules, which appear to be hydrophobic, are based on hydrophobic interactions in binding to PLO. When the key conserved point in the PLO protein is mutated from a hydrophobic amino acid to an polar amino acid, the binding energy will be decreased, resulting in repulsion of cholesterol molecules in response to hydrophilic amino acids. Comparing with the amino acids in the original site of the protein, the change in binding energy of N139K is greater than that of F240. Therefore, we boldly speculate that this may also contribute to the complete disappearance of pore-forming ability of rPLO N139K. These results are consistent with the result of pore-forming ability in the present study. However, the mutation of rPLO N139K at residue 139 from asparagine to lysine, a polar amino acid with strong hydrophilicity, can form hydrogen bonds and lose its ability to bind small hydrophobic molecules. This is why the mutation effects of rPLO N139K and rPLO F240A are both beneficial. However, this explanation cannot describe why the mutations of N139K and F240A also reduced pore-forming ability and other toxicological characteristics. Furthermore, the interaction between protein and small molecules is also affected by the structure of the key point(s) of the protein, which is the extent of matching between the small molecule and the protein “pocket” structure when small molecules attempt to reach in and stick inside. Asparagine and phenylalanine are hydrophobic groups compared to lysine and alanine, respectively, which have no mutual repelling force for small hydrophobic molecules to approach the “pocket” but provide hydrophobic mutual interaction.

We observed that the mutations of residues 139 and 240 of PLO uncoupled membrane binding and deformed large oligomers from the structural transitions. These actions are necessary for the assembly of the pore complex (13). In essence, the monomer structure of these mutants seems comparatively untransformed from that of the PLO monomer. Therefore, this study found for the first time the influence of the conserved amino acids Asn-139 and Phe-240 on the conformation and pore-forming activity of PLO and explain in detail the reasons for the changes in pore-forming activity and correlate pore formation with the conformation of the toxin. Additionally, some studies have reported that loss of indispensable cation-π interaction owing to replacement a residue in domain 1, associated with the conformational rigidity of domain 3, was found to be the major contributor to the loss of pore-forming ability of Ply-NH, which is another important member of the CDC family (34). Interestingly, these subtle substitutions accomplished by a single-residue mutation are adequate to efficaciously limit the mutant PLOs from performing their functions. From an evolutionary perspective, this might be more beneficial, as new traits can appear in a short period without inducing considerable changes in the protein structure, by which absence of pore-forming ability of CDC members supplies an intracellular survival superiority to bacteria to escape antibacterial autophagy (34).

*T. pyogenes* usually causes inflammatory diseases in different species, including abortion, arthritis, endocarditis, mastitis, pneumonia, and osteomyelitis (30). PLO, as the main weapon of *T. pyogenes*, performs sabotage tasks, which include disruption of cell barriers and evasion of host immune responses (2). Such a minor change in PLO caused such a major impact on its functions. TEM and real-time PCR results of mouse tissues showed that rPLO activated the inflammasome cytokines, inflicted striking structural damage, and induced inflammatory cell infiltration into mouse tissues. Recent studies clarified that activation of inflammatory cytokines is related to K^+^ efflux, which can reduce the activation of the Nod-like receptor family pyrin domain containing 3 (NLRP3) pathway (33, 40). More reports indicate that PLO can upregulate inflammatory cytokine levels in different cells (41–43). However, compared to rPLO, the ability of rPLO F240A and rPLO N139K to activate inflammatory cytokines is significantly weakened, which may just be related to total or partial loss of pore-forming ability. Moreover, compared to these mutants, PLO has a better chance of being recognized by the innate immune system in mice, followed by mobilization of immune cells. We speculate that this difference may come from substitution of different amino acids to make the membrane-binding ability undergo essential changes.

To conclude, the F240A and N139K mutations severely influenced intra- and extramolecular interactions in cholesterol binding and oligomerization in PLO toxin. Based on our data, we hypothesize that the F240A and N139K mutations impair the optimal interaction mechanism between PLO and cholesterol-dependent membranes and abolish the key components in triggering fractional conformational change. Our studies elaborate a detailed analysis of the function and mechanism of PLO pore formation, as a special member of the CDC family of proteins widely existing in nature. Our data also provide more of a theoretical basis and practical significance for the future study of similar toxins and bacteria.

## MATERIALS AND METHODS

### Cell culture.

The human epithelial carcinoma HeLa cell line (ab260075, Abcam) and primary bovine endometrial epithelial cells (BEECs) were respectively cultured in Dulbecco’s modified Eagle’s medium (DMEM)/high glucose (SH30022.01, Cytiva) and DMEM (SH30023.01, Cytiva) supplemented with 10% fetal bovine serum (FBS; 10099141, Gibco) and penicillin/streptomycin (15140122, Gibco) at 37°C and 5% CO_2_. Primary BEECs were obtained from three Holstein cows for cell culture after sacrifice and were previously isolated, purified, and preserved in our laboratory (44).

### Bacterial strains, plasmids, and chemicals.

PLO genes and mutant variants of PLO were cloned into pET32a (Addgene) as described previously (17, 28, 45). Both mutants, rPLO N139K and rPLO F240A, were produced based on the same method of generating rPLO protein. All enzymes and chemicals were purchased from Sigma Chemical Co. (St. Louis, MO), Solaibio Life Sciences (Beijing, China), TransGen Biotech (Beijing, China), and Beyotime Biotechnology (Shanghai, China) unless otherwise specified.

### Generation and purification of toxin.

Fusion PCR via overlap sequences was used for generating the individual amino acid substitutions in PLO, and sequence verification was performed at Sangon Biotech (Shanghai) by DNA sequencing. The expression and purification of rPLO and its mutants containing an amino-terminal His tag from Escherichia coli were conducted as described previously (1, 23, 45). Briefly, freshly transformed colonies of E. coli DH5α (CD201, TransGen) containing pET32a were grown in Luria Bertani (LB) broth containing 100 μg/mL ampicillin at 37°C on a shaker incubator for 12 h. Five milliliters of the culture was added to 500 mL of LB broth and shaken at 37°C until the optical density at 600 nm (OD_600 nm_) reached 0.6. The addition of 200 μM isopropyl-l-thiogalactopyranoside (IPTG) promoted protein expression for an additional 20 h with shaking at 16°C at 120 rpm. The cell pellet was resuspended with ice-cold phosphate-buffered saline (PBS; P1020, Solarbio) three times after centrifugation at 5,000 rpm for 10 min at 4°C and sonicated to disintegration for 20 min in 5 mL of PBS on ice. The supernatant was applied to the ProteinIso nickel-nitrilotriacetic acid (Ni-NTA) resin (DP101, TransGen) according to the manufacturer’s instructions. After washing with 10 volumes of equilibration buffer, proteins were eluted by gradient dilutions of imidazole from 50 to 300 mM in equilibration buffer. Subsequently, the protein was centrifuged using a 30-kDa molecular weight ultrafilter tube at 2,500 × *g* for 5 min at 4°C several times to remove the imidazole and equilibration buffer with PBS. After the protein concentration was measured by Nano Drop (ND-ONE-W, Thermo Fisher), the purity was then detected by SDS-PAGE. Both of the PLO mutants were expressed and purified with the same protocol.

### Hemolysis activity.

Five milliliters of fresh sheep blood was washed and resuspended in a sterilized 50-mL centrifuge tube three times with preiced PBS (pH 7.4) by centrifuging at 4°C at 2,500 rpm for 10 min to obtain a 2% red blood cell (RBC) suspension diluted with PBS. The initial concentrations of recombinant PLO (rPLO) protein and PLO mutants were adjusted to 100 μg/mL, and continuous 2-fold dilutions were performed. Fifty microliters of rPLO and the mutants were incubated with 50 μL of 2% sheep red blood cells (SRBCs) together, respectively, at 37°C for 30 min. A PBS control group and a whole-blood control group were processed by the same incubation. After 30 min, one hemolytic unit (1 HU) of rPLO was determined and calculated, comparing with the mutants, which are lacking structure or function.

### Membrane-binding ability.

Five milliliters of fresh sheep blood was added to 3 times the volume of preiced PBS in a 50-mL centrifuge tube and washed 3 times at 4°C at 2,000 rpm by centrifuging for 5 min. The supernatant was carefully removed, and 45 mL of deionized water was added to the red blood cells. After evenly pipetting and fully swelling at 4°C for 4 h, the red blood cells were centrifuged at 4°C at 5,000 rpm for 10 min. The pellet was washed 3 times with deionized water and resuspended in 5 mL of PBS to obtain an RBCM suspension. The initial concentrations of the recombinant proteins of rPLO and mutants were adjusted to 100 μg/mL; 500 μL of the recombinant proteins and equal volume of the RBCM suspension were incubated at 37°C for 30 min as described previously (37). Briefly, the supernatant and precipitate were separated by centrifugation at 12,000 rpm for 10 min at 4°C. The supernatant was applied by trichloroacetic acid (A11156, Thermo Fisher) overnight for separating the components. The precipitates were resuspended in NaOH (S111498, Aladdin). Equal volumes of all samples were separated by sodium dodecyl sulfate-polyacrylamide gel electrophoresis (SDS-PAGE) on 12% gels and transferred onto a polyvinylidene fluoride (PVDF; IPVH00010, Merck) membrane. The mouse anti-His tag monoclonal antibody was used for Western blotting.

### Oligomerization determination.

Lecithin (0.281 g) and cholesterol (0.091g; mass ratio of 3:1) were dissolved into 20 mL of ether and slowly dropped to 20 mL of PBS at 30°C with thorough stirring for 10 min. The mitigation solution was heated to 55°C for 1.5 h. After the ether was completely evaporated, the solution was ultrasonic treated for 20 min and filtered with a 0.45-μm filter to obtain the liposomes. rPLO (120 μL) and mutant proteins were mixed with equal volume of liposomes, respectively, and incubated at 37°C for 2 min, 5 min, 10 min, and 20 min. All samples with 5 × loading buffer were boiled in a water bath. Twenty-five-milliliter samples were loaded in 8% SDS-PAGE gels and transferred to PVDF membranes (75 mA, 120 min). Western blotting was performed with a mouse His-tagged primary antibody (1:5,000 dilution) and a horseradish peroxidase (HRP)-conjugated goat anti-mouse IgG secondary antibody (1:5,000 dilution).

### Liposome preparation.

Liposomes were synthesized as previously described (40). Briefly, cholesterol (23%) and lecithin (67%) were mixed in a solution of chloroform and methanol (2:1, vol/vol). The mixture was then dried in a rotary evaporator under vacuum at 40°C for 3 h to mimic the mammalian cell membrane (46). Liposomes were loaded with methylene blue dye (75 μg/mL). The methylene blue dye was encapsulated in the lumen of the liposome, resulting in leakage of the dye induced by PLO and the mutants, which allowed us to explore the ability of those proteins to form pores in the liposome membrane. Liposomes were exposed to rPLO, mutants, PBS, or buffer. The dye released after pore formation in liposomes was captured by a cation exchange resin Dowex (15 mg/well). A microplate reader (Bio-Rad) was used for residual dye measurement (OD_600_; 590-nm wavelength).

### Cytotoxicity assay.

A lactate dehydrogenase (LDH) release assay kit was used for measurement of the cytotoxic effect of PLO and PLO variants as per the manufacturer’s instructions.

### Ethics statement.

All mouse experiments were performed in accordance with the Guidelines for Laboratory Animal Use and Care from the Chinese Centers for Disease Control and Prevention and the Rules for Medical Laboratory Animals (1998) from the Chinese Ministry of Health. The protocol, AW03111202-1-1, was approved by the Animal Ethics Committee of the China Agricultural University. CD1 mice of 6 weeks of age were randomly assigned to four groups for infection experiments individually. Each group contained 8 mice.

### Animal infection experiment.

A total of 32 6- to 8-week-old CD1 mice were randomly divided into four groups and received 300 μL of PBS or 200 μg of rPLO, rPLO N139K, or rPLO F240A in 300 μL, respectively, via intramuscular administration on days 0, 3, and 5. All mice were humanely sacrificed on day 3 after the third injection, and muscle tissues at the injection site were taken for comparison of animal histopathological changes caused by rPLO and the rPLO mutants via real-time PCR and hematoxylin and eosin (H&E) staining.

### Transmission electron microscopy (TEM).

Freshly collected animal muscle tissues with different protein treatments were fixed in 3% glutaraldehyde (pH 7.4) at room temperature for 48 h and processed with TEM procedures. TEM images were acquired with an H-7500 transmission electron microscope (Hitachi, Tokyo, Japan).

### Histology analysis.

Harvested mouse tissues were fixed in 10% formaldehyde solution. The samples were embedded with paraffin, sectioned, and stained with hematoxylin and eosin (H&E). The stained tissue sections were visualized with an OVT DVL1400 microscope under ×20 and ×40 magnification, and images were captured with OVT View 3.7 software.

### Cytokine measurements.

Total RNA was extracted with TRIzol reagent (Invitrogen, Carlsbad, CA) from four treated mouse muscle tissues. The concentration of RNA was measured by NanoDrop (Thermo Fisher, Waltham, MA, USA). RNA was reverse transcribed into first-strand cDNA for real-time PCR. Mouse interleukin-1β (IL-1β) and interleukin-18 (IL-18) expression levels in muscle from differently treated mice were measured. To normalize the cycle threshold (*C_T_*) values, expression of the hypoxanthine phosphoribosyl-transferase housekeeping gene was evaluated. Cytokine measurement data are displayed with the method of 2^−ΔΔ^*^CT^* as fold change.

### Structural modeling analysis and molecular docking.

The PLO protein amino acid sequence was obtained from the National Center for Biotechnology Information (NCBI) website, and the cholesterol compound structure came from the PubChem database. The PLO sequence is highly conserved in standard and clinically isolated *T. pyogenes*. The three-dimensional (3D) *in silico* models of the monomeric form of the rPLO and homology-based mutant molecules were generated using the Phyre^2^ server (http://www.sbg.bio.ic.ac.uk/phyre2/html/page.cgi?id=index) (47). The predictions of protein ligand-binding sites were operated by COACH analyses from the Zhang Lab server (48). The protein structures were imported into AutoDock Tools software (1.5.6 version) before the deletion of water molecules and the addition of hydrogen atoms and saved as a PDBQT file. The small-molecule compounds were imported into the same software, and the atomic charges were added, atomic types were assigned, and all flexible bonds were set to be rotatable by default after the water molecules were deleted. All docking operations used AutoDock Tools 1.5.6 software. In the calculation process, a 40 × 40 × 40 lattice module with 0.375 intervals was used to completely cover the specified residues. A Lamarckian genetic algorithm was used for the molecular docking calculation, and the final docking structure was evaluated based on the binding free energy. The hydrophobicity of the protein was analyzed by HeliQuest analysis (https://heliquest.ipmc.cnrs.fr). All protein structure models and docking results were visualized using PyMol 2.3 software (5).

### Statistical analysis.

The statistical analysis was performed in GraphPad Prism version 9. A one-way analysis of variance (ANOVA) test was used, and multiple corrections were applied by Tukey’s honestly significant difference *post hoc* test. Data are presented as mean ± standard error of mean value (SEM; *n* = 3 or 6). A *P* value of <0.05 was considered statistically significant.
