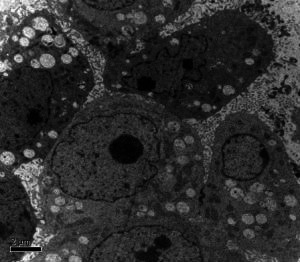# Correction for Liu et al., “Single Point Mutation and Its Role in Specific Pathogenicity to Reveal the Mechanism of Related Protein Families”

**DOI:** 10.1128/spectrum.03663-23

**Published:** 2023-12-06

**Authors:** Ning Liu, Xue Wang, Qiang Shan, Shuxian Li, Yanan Li, Bingxin Chu, Jiufeng Wang, Yaohong Zhu

## AUTHOR CORRECTION

Volume 10, no. 5, e00923-22, 2022, http://doi: 10.1128/spectrum.00923-22. Page 8, Fig. 5D : A previously used image was inadvertently used. The bottom panel in the “Untreated” column should appear as shown below. The change does not affect the results and conclusions of the paper.

**Figure:**